# Cancer mortality in former East and West Germany: a story of unification?

**DOI:** 10.1186/s12885-017-3086-y

**Published:** 2017-02-02

**Authors:** Daniel Medenwald, Dirk Vordermark, Christian T. Dietzel

**Affiliations:** 1 Department of Radiation Oncology, University Hospital Halle (Saale), Ernst-Grube-Str. 40, 06120 Halle (Saale), Germany; 2 Institute of Medical Epidemiology, Biostatistics and Informatics, Martin-Luther-University Halle-Wittenberg, Magdeburger Str. 8, 06112 Halle (Saale), Germany

## Abstract

**Background:**

Health and social conditions vary between West and East Germany.

**Methods:**

We analyzed annual mortality data of all recorded deaths caused by lung, colorectal, breast and prostate cancer in Germany as they are published by the Federal Bureau of Statistics (FBS) encompassing the period 1980–2014 for former West Germany (WG) and 1990–2014 for former East Germany (EG). To compare East and West Germany we computed the ratio of the mortality rates in both parts (mortality rate ratio, MRR, <1 indicates a lower mortality in EG). Forecasting methods of time series analyses were applied (model selection based on the Box/Jenkins approach) to predict 5-year trends until 2019.

**Results:**

Lung cancer: In women mortality rose in both regions (WG: +2.8%, 1991–2014, EG: +2.2%, 1990–2014). In men mortality in WG declined between −2.1% and −1.2%, and by −2.7% (1993–2009) in EG which was followed by a plateau. Colorectal cancer: A decline was found in both WG (−3.1%, 1993–2014) and EG women (−3.8%, 1993–2008 and −2.0%, 2008–2014). A decline in EG men since 1992 (−0.9%, 1992–1997 and −2.3%, 1997–2014) mirrors the development in WG (−2.6%, 1995–2014). Breast cancer: Constant mortality decline in WG after 1996. In EG a decline (−2.4%, 1992–2007) was followed by a plateau with an MRR <1 (1990–2014). Prostate cancer: In WG a decline (−3.4%) came to a hold after 2007, while there was a constant decline of 1.5% in EG. The forecast indicated that mortality of colorectal/lung cancer in men and breast cancer reaches a plateau in future years.

**Conclusion:**

Courses of mortality were similar between East and West, while existing differences are likely to remain in the near future.

**Electronic supplementary material:**

The online version of this article (doi:10.1186/s12885-017-3086-y) contains supplementary material, which is available to authorized users.

## Background

It is a great achievement that cancer mortality has decreased in Europe since the early 1980s [1]. Unfortunately cancer survival is not spread evenly over the continent. While 5-year age and cancer site case-mix standardized relative survival rate (ACRS) appears to be lowest in Bulgaria for both men (32%) and women (47%), Austrian men (53%) and Icelandic women (61%) currently have the highest chances of survival [2]. Overall there is an approximately 9% lower probability of 5-year survival in Eastern Europe compared to Central Europe.

One reason for this discrepancy might be found in differing socioeconomic welfare and health funding [3, 4]. The reunification of Germany in 1990 led to the establishment of a markedly different political and economic system in the East, influencing a renewal in general health care.

While cancer survival for a variety of sites was poorer in the German Democratic Republic (GDR, East Germany) compared to the Federal Republic of Germany (FRG, West Germany) in the early 1980s, an ongoing improvement in Eastern Germany has resulted in more favourable treatment outcomes during the last years [5].

*Jansen et al.* estimated the 5-year age-standardized relative survival rate for the 25 most common cancer localizations using data from 11 German cancer registries for 2002–2006 [5]. Finding very similar survival rates for 20 of the analysed cancer sites (differences < 3%), the authors concluded that there was a rapid closure of the former survival gap between East and West Germany. However, there were obvious advantages for patients suffering from cancer of the gallbladder, oesophagus, oral cavity, or skin melanoma in the West and leukaemia in the East.

An interesting question not answered by recent publications is whether Eastern Germany will eventually overtake the rest of the country regarding cancer survival. The goal of this study is to model future projections of mortality by analysis and comparison of both West and East Germany using the method of time series analysis.

## Methods

### Statistical analysis

#### Mortality data

The annual mortality data of all recorded deaths in Germany published by the Federal Bureau of Statistics (FBS) was taken into account (Gesundheitsberichterstattung des Bundes, www.gbe-bund.de). The available period encompasses the time between 1980 and 2014 for former West Germany and 1990 to 2014 for former East Germany. We computed sex-specific mortality rates (per 100,000 inhabitants) after age standardization (world standard population) separately for East and West Germany. A direct age standardization approach with five-year age intervals was used for each separate cancer. In all analyses distinguishing between both areas of Germany, the area of Berlin was excluded. This was due to a divided Berlin; one part belonged to West Germany and the other to the former East Germany. Mortality data released by the FBS referred to the unified area of Berlin without differentiating between its former parts. Eventually, the area of Berlin was considered in an additional analysis which is presented in the supplement.

We focused on the three most common types of cancer-related deaths in Germany: lung cancer, colorectal cancer, and prostate cancer in men and breast cancer in women (Gesundheitsberichterstattung des Bundes, www.gbe-bund.de).

### Time series analysis and forecasting

Time series analysis and autoregressive integrated moving average (ARIMA) were used for statistical analyses. Model identification was based on the algorithm introduced by Box and Jenkins [6, 7]. Details of model identification and parameter estimation, including the order of the selected ARIMA models, are provided in the Additional file 1. Based on the most parsimonious ARIMA model (requiring the least moving average and autoregressive terms) that was in accordance with the Box/Jenkins approach, we applied the “predict” function in R which uses an approach based on the Kalman filter to forecast future mortality rates until the year 2019. This function also displayed their respective 95% confidence intervals.

To compare East and West Germany we computed the ratio of the mortality rates in both areas (referred to as mortality rate ratio, MRR). Both mortality rates and MRRs underwent log transformation during statistical analyses but were eventually re-transformed for illustrative purposes. Similar to age-adjusted mortality, we performed a time series analysis to forecast future MRR and a joinpoint analysis considering the historical MRR data.

### Joinpoint analysis

In addition to the time series analysis, we examined the linear trends of the time series by means of a regression based approach with time as the explanatory variable. A joinpoint analysis was performed to identify time points when alterations in the linear trend appear in the time series [8]. We performed a permutation test with 2000 permutations allowing for a maximum of four join points (trend alterations). Joinpoint analyses were performed using the software package Joinpoint 4.2.0.2 developed by the Statistical Research and Applications Branch, National Cancer Institute (NCI). The software can be complimentarily downloaded from the NCI webpage (http://surveillance.cancer.gov/joinpoint).

### Internal validation

An internal validation was performed to assess the possible bias and the precision of the five year forecast models by comparing the predicted with the observed mortality from 2010–2014.

### Sensitivity analysis

To estimate the impact of a possible disturbance in the reported mortality by rearrangements in the coding system during the transition period, we performed a sensitivity analysis where the early years from 1990–1995 were excluded from the East German mortality data.

All further statistical analyses and data management were performed using R version 3.1.2 (http://www.R-project.org) [9].

## Results

### Lung cancer

**Fig. 1:**
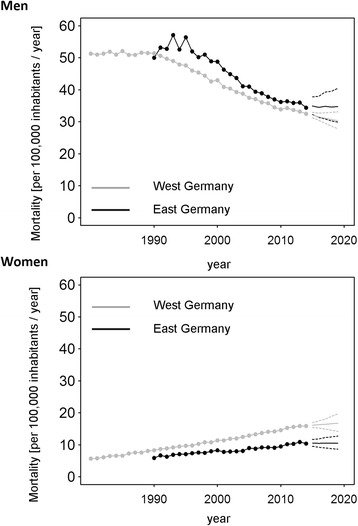
Time series of annual mortality rates for death from lung cancer in Germany. Black: East Germany; Grey: West Germany. Solid lines represent the estimates of the forecast with the respective 95% confidence intervals (dashed lines)

**Fig. 2:**
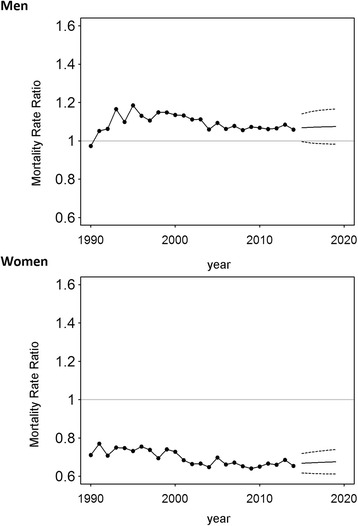
Ratio of annual mortality rates in East and West Germany for death from lung cancer in Germany. The mortality rate ratio was computed as the ratio of the annual age-standardized mortality in East Germany to that in West Germany

We estimated a constant growth of lung cancer mortality for German women overall. The site-specific rise was higher for West German women (Table 2) with an annual increase of 2.8% (95% CI: 2.7%, 2.9%, period 1991–2014) than for East German women (Table 1) at 2.2% (95% CI: 2.0%, 2.4%, period 1990–2014). Trend analyses predicted a plateau in mortality for East German women for the period after 2014, whereas mortality is predicted to grow only minimally in West Germany (Fig. 1). Between 1990 and 2014 MRRs were below the equivalence value of one, indicating that lung cancer specific death was less common in East Germany as compared to West Germany (Fig. 2). In women, it was estimated that the MRR decreased (−0.6; 95% CI: −0.8, −0.4, Table 3) constantly during the observation period (increasingly lower mortality in East Germany when compared to West Germany). The difference in mortality is predicted to stay constant for upcoming years as indicated by confidence intervals considerably and constantly below the equivalence value.

**Table 1:** Annual percentage mortality change in East Germany with 95% confidence intervals

East				
Men				
		Slope 1	Slope 2	Slope 3
Lung	Year	1990–1993	1993–2009	2009–2014
	Estimate	4.4 [0.7, 8.2]	−2.7 [−3.0, −2.4]	−1.1 [−2.6, 0.5]
Prostate	Year	1990–1993	1993–2014	-
	Estimate	11.8 [5.0, 19.0]	−1.5 [−1.8, −1.2]	-
Colon/Rectum	Year	1990–1992	1992–1997	1997–2014
	Estimate	10.9 [3.7–18.5]	−0.9 [−2.9, 1.3]	−2.3 [−2.5, −2.1]
Women				
Lung	Year	1990–2014	-	-
	Estimate	2.2 [2.0–2.4]	-	-
Breast	Year	1990–1992	1992–2007	2007–2014
	Estimate	4.7 [−3.0, 13.1]	−2.4 [−2.7, −2.0]	0.4 [−0.6, 1.5]
Colon/Rectum	Year	1990–1993	1993–2008	2008–2014
	Estimate	4.2 [−0.1, 8.7]	−3.8 [−4.1, −3.4]	−2.0 [−3.3, −0.6]

Estimates were computed using a Joinpoint analysis with 2000 permutations

Lung cancer mortality in West German men declined about 2.1% (95% CI: 2.1%, 2.2%) per year between 1991 and 2009, and 1.2% (95% CI: 0.6%, 1.7%) between 2009 and 2014 (Table 2). This process is expected to lose momentum through 2019 (Fig. 1). However, lung cancer mortality in East German men increased about 4.4% (95% CI: 0.7%, 8.2%) annually between 1990 and 1993, followed by a decline of 2.7% (95% CI: 2.4%, 3.0%) for the period 1993–2009 and a plateau phase with only a minor, insignificant decline during 2009–2014 (Table 1). The trend analysis revealed no further decrease in mortality until 2019 (Fig. 1) for men in East Germany. The MRR has remained above one since 1991, although the lower 95% confidence limits are below one with a value of 0.99 for future predictions. This describes a persistently higher site-specific mortality in East Germany (Fig. 2). The joinpoint analysis revealed a considerable percentage increase in the MRR from 1990–1993 (5.6; 95% CI: 2.3, 9.0, Table 3), equivalent to an increasingly disadvantageous situation in East Germany versus West Germany. This development was reversed in subsequent years by a slowly decreasing MRR, or an increasingly advantageous mortality in East Germany as compared to West Germany.

**Table 2:** Annual percentage mortality change in West Germany with 95% confidence intervals

West					
Men					
		Slope 1	Slope 2	Slope 3	Slope 4
Lung	Year	1980–1991	1991–2009	2009–2014	-
	Estimate	0.0 [−0.2, 0.2]	−2.1 [−2.2, −2.1]	−1.2 [−1.7, −0.6]	-
Prostate	Year	1980–1995	1995–2006	2006–2014	-
	Estimate	0.7 [0.5, 1.0]	−3.4 [−3.9, −3.0]	−0.6 [−1.2, −0.0]	-
Colon/Rectum	Year	1980–1995	1995–2014	-	-
	Estimate	−0.2 [−0.4, −0.0]	−2.6 [−2.7, −2.4]	-	-
Women					
Lung	Year	1980–1991	1991–2014	-	-
	Estimate	4.0 [3.7, 4.3]	2.8 [2.7, 2.9]	-	-
Breast	Year	1980–1987	1987–1996	1996–1999	1999–2014
	Estimate	1.5 [0.9, 2.0]	−0.2 [−0.7, 0.3]	−3.4 [−7.4, 0.8]	−1.6 [−1.8, −1.4]
Colon/Rectum	Year	1980–1993	1993–2014	-	-
	Estimate	−0.8 [−1.0, −0.6]	−3.1 [−3.2, −3.0]	-	-

Estimates were computed using a Joinpoint analysis with 2000 permutations

**Table 3:** Annual percentage change in the ratio of East to West German cancer mortality with 95% confidence intervals between 1990 and 2014

Men			
		Slope 1	Slope 2
Lung	Year	1990–1993	1993–2014
	Estimate	5.6 [2.3, 9.0]	−0.4 [−0.6, −0.3]
Prostate	Year	1990–1992	1992–2014
	Estimate	17.5 [3.0, 34.0]	1.0 [0.7, 1.3]
Colon/Rectum	Year	1990–1992	1992–2014
	Estimate	10.8 [4.3, 17.6]	0.3 [0.2, 0.5]
Women			
Lung	Year	1990–2014	-
	Estimate	−0.6 [−0.8, −0.4]	-
Breast	Year	1990–1996	1996–2014
	Estimate	−0.4 [−0.9, −0.0]	1.5 [0.2, 2.8]
Colon/Rectum	Year	1990–1992	1992–2014
	Estimate	6.2 [−4.3, 17.8]	−0.3 [−0.5, −0.0]

Results refer to the relative change in the mortality rate ratio as computed by means of a joinpoint procedure

### Colorectal cancer

**Fig. 3:**
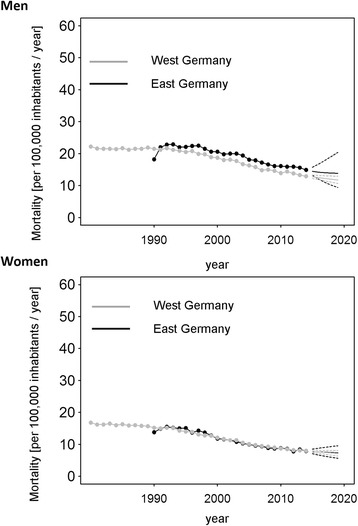
Time series of annual mortality rates for death from colorectal cancer in Germany. Black: East Germany; Grey: West Germany. Solid lines represent the estimates of the forecast with the respective 95% confidence intervals (dashed lines)

For East German women, an initial increase in mortality (4.2%, 95% CI: −0.1%, 8.7%, 1990–1993) was followed by an enduring decline of 3.8% (95% CI: 3.4%, 4.1%, 1993–2008, Table 1) and 2.0% (95% CI: 0.6%, 3.3%, 2008–2014). For West German women, we detected a constant decrease of 3.1% (95% CI: 3.0%–3.2%, 1993–2014, Table 2). The trend analysis predicted a similar annual decline in West German females until 2019 (Fig. 3).

**Fig. 4:**
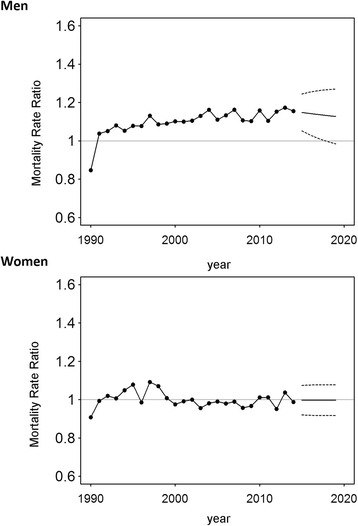
Ratio of annual mortality rates in East and West Germany for death from colorectal cancer in Germany. The mortality rate ratio was computed as the ratio of the annual age-standardized mortality in East Germany to that in West Germany

Between 1992 and 2014 MRR undulated consistently around the equivalence value, illustrating a comparable colorectal cancer mortality for East German and West German women during this period which is expected to stay constant until 2019 (Fig. 4). The MRR increased by 6.2% between 1990 and 1992, with almost no change in the ratio of East German to West German cancer mortality observed in later years (Table 3).

The decrease in colorectal mortality in West German males was unaltered between 1995 and 2014 at 2.6% (95% CI: 2.4%, 2.7%) annually (Table 2). In East German men, a rise during the early years after reunification (10.9% 95% CI: 3.7%, 18.5%, 1990–1992) was followed by a slower decrease of 0.9% (95% CI: −1.3%, 2.9%, 1992–1997) and a stronger yearly decline of 2.3% (1997–2014, Table 1). The MRR remained >1 between 2009 and 2014, indicating a survival benefit in West German males (Fig. 4). For East German men, the MRR increased by an estimated relative change of 10.8% (1990–1992), yet decelerated from 1992 until 2014 (Table 3). It was predicted that this trend would continue, and the mortality rate ratio will undergo a slow decrease until 2019 (Fig. 3).

### Breast cancer

**Fig. 5:**
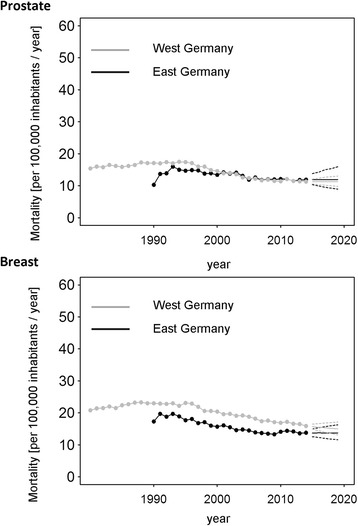
Time series of annual mortality rates for death from breast and prostate cancer in Germany. Black: East Germany; Grey: West Germany. Solid lines represent the estimates of the forecast with the respective 95% confidence intervals (dashed lines)

**Fig. 6:**
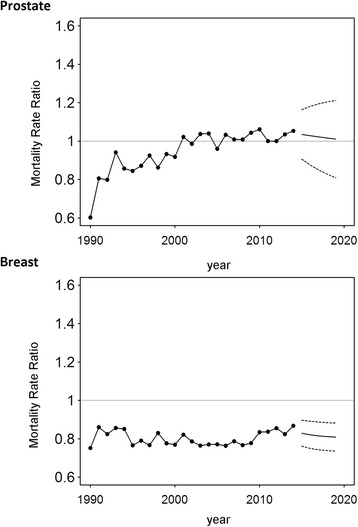
Ratio of annual mortality rates in East and West Germany for death from breast and prostate cancer in Germany. The mortality rate ratio was computed as the ratio of the annual age-standardized mortality in East Germany to that in West Germany

The analysis of breast cancer mortality in East Germany in the aftermath of reunification highlights a contrasting picture (Table 1). After a noticeable annual decrease of 2.4% (95% CI: 2.0%, 2.7%) between 1992 and 2007, we estimated a minor rise of 0.4% (95% CI: −0.6%, 1.5%) between 2007 and 2014. Breast cancer mortality was lower in the former GDR for the entire time period (MRR <1, 1990–2014; Figs. 5 and 6). Since 1996, increasingly disadvantageous cancer mortality was observed in East German women as compared to West German women (Table 3). The trend analysis predicted a continuing survival benefit in East Germany until 2019, though mortality in West Germany is expected to further decline (Fig. 6).

### Prostate cancer

Mortality from prostate cancer was found to decline slightly during the observational period of 1995–2006 by 3.4% per year (95% CI: 3.0%, 3.9%) in West Germany (Table 2) but came to a halt afterwards. In East Germany a weaker decline of 1.5% (95% CI: 1.2, 1.8%, 1993–2014) per year endured until the end of the observational period (Table 1). The forecast indicated virtually no change in mortality in both parts of Germany, though the wide confidence intervals need to be considered (Fig. 5).

When comparing East and West Germany by means of MMRs, it was found that mortality was slightly higher in West Germany until the year 2000. As in the previous analyses, the MRR rose considerably in the first years after reunification (Table 3). The successive equivalence of both parts in terms of mortality from prostate cancer is expected to sustain in the upcoming years, although confidence intervals are again wide (Fig. 6).

### Internal validation

When we performed an internal validation (Additional file 2: Figure S1 of the supplement), the bias estimated as the mean of the difference between the predicted and observed value (forecast error) was −2.1% (95% CI: −3.3, −0.9). Thus, the presumed models tend to underestimate the mortality by 2% on average. The precision of the predicted mortality decreased in the 95% prediction intervals, except for lung cancer in East German women. In our example, the prediction intervals indicate a 95% chance that the five-year forecast has an interval between 88.6% and 107.1% of the observed value. When female lung cancer mortality in East Germany is excluded, the interval narrows to 93.5% and 104.1% respectively.

### Sensitivity analysis (Additional file 3: Figure S2, Additional file 4: Figure S3, Additional file 5: Figure S4, Additional file 6: Figure S5, Additional file 7: Figure S6, Additional file 8: Figure S7) and Berlin

In the sensitivity analysis, excluding the mortality reported in early years, we found comparable point estimates of the forecast but smaller confidence intervals when death from colorectal cancer in men and the MRR of prostate cancer was considered. The slope changed towards a steeper decrease only in the case of lung cancer mortality in men when compared with the entire time series.

In regards to the results of Berlin, the mortality from the remaining German population is mirrored by this population, both in magnitude and trend (Additional file 9: Figure S8, Additional file 10: Figure S9, Additional file 11: Figure S10, Additional file 12: Table S1).

## Discussion

At first glance, the steep rise in cancer mortality after the reunification of East Germany seems implausible. General health care was expected to have improved during this period of time in East Germany, and the physiology of cancer renders fast changes in mortality impossible. The actual cause for this might be based on the different coding systems that the GDR and FRG used in order to register causes of death [10, 11]. The structure of the GDR´s coding system is supposed to have led to underreported cancer mortality before 1990 due to subjective evaluation of the leading cause of death by the certifying doctor. Thus, the adoption of West Germany’s coding practice led to a more precise evaluation. The effect seems to be more pronounced in East German men, which is likely affected by the overall higher cancer mortality in this group.

Though there was a trend of young, healthy, and well-educated East Germans migrating to West Germany especially in 1989/1990 (“selective migration”), the influence of this population shift on overall mortality remains uncertain [11, 12]. For all cancer types, migration might be a factor that contributes to the observed results. However, in this case migration should bias towards cancer. That is, conditioning on age, people that were less likely to get cancer should have been more likely to migrate to West Germany. We expected a sharp decline in cancer mortality after reunification, which is not supported by the data. The typical late age at the time of diagnosis made it unlikely that the vast majority of potential cases moved to either part of Germany. This line of thought is more comprehensively followed in Luy et al.

### Lung cancer

Smoking is undeniably the key contributor to lung cancer mortality worldwide [13].

An analysis of German microcensus data determined the sex-specific proportion of current smokers from 1989 to 2009 [14]. While the rates for females remained stable with 17.9% in 1989 and 18.9% in 2009, the number of male smokers dropped significantly from 36.7% to 27.5%. These findings of increasing mortality in women and a decreasing trend in men might be closely linked to the described differences in smoking behaviour.

Varying tobacco consumption is also a plausible explanation for the reversing mortality difference among East German and West German women as stressed in a recent publication by Myrskylä et al. [15]. The authors argued that smoking, which is more common among West German women, led to a lower decline in smoking-attributable mortality in this population as compared to their East German counterparts. Lung cancer showed a more advantageous mortality in East Germany, while mortality rates of other cancer types were of comparable magnitude in both areas. Lower mortality in East Germany was especially present in females between 50 and 64 years of age.

Our data matches with a prognosis by Malvezzi et al. that calculated age-standardized mortality rates for the European Union [16]. Between 2009 and 2015 they predicted a fall of 9.1% for males, but described a rise of 9.2% for females.

### Colorectal cancer

A declining mortality from colorectal cancer was found in our data. Using the WHO mortality database, Bosetti et al. [17] determined an annual percent change in men of −0.6% (1992–2003) and −2.0% (2003–2007), while a continuous decline of 1.6% was detected in women. Overall mortality in females was lower (10.51/100.000 in 2007) than in males (17.35/100.000).

A triad of improved early detection of disease, new treatment options for more advanced stages, and a healthier lifestyle in general may have driven the results.

The use of faecal occult blood testing and colonoscopies as screening techniques allowed early detection of adenomatous polyps as possible malignant precursors and early stage tumours with a favourable prognosis [18, 19]. Tremendous therapeutic changes like new approaches in surgery, the utilization of neoadjuvant chemoradiotherapy for rectal carcinoma, and adjuvant chemotherapy protocols contributed to higher cure rates [20–23].

A health conscious diet is an important aspect in the prevention of colorectal cancer [24]. This might be a feasible explanation for the geographical differences regarding mortality. Mensink et al. described a lower consumption of cereals and leafy vegetables in East Germans years after the reunification [25].

Though people should have been examined for colorectal cancer starting at age 40 in the former GDR, 29.2% of cases with colon cancer and 24.7% of cases with rectal cancer were diagnosed at stage IV in 1987. This is a modest rise when compared to 1977 (24.3% and 19.7%) and could explain the increase that was observed in 1990/1991 in East Germany [26].

### Breast cancer

Numerous factors contributed to the general mortality decrease during recent decades throughout Germany for breast cancer patients.

First, there was tremendous improvement regarding treatment strategies including the introduction of new systemic therapy regimens (especially hormone therapy and taxane-based chemotherapy), breast-conserving surgery, and adjuvant radiotherapy [27–30].

The establishment of a nationwide screening program in 2005 using x-ray mammography is expected to have led to a significant rise in the detection of early stage disease, although its effects are unlikely to have contributed considerably to our findings. A synopsis of European incidence-based mortality (IBM) studies investigating the efficiency of breast cancer screening revealed a mortality reduction of about 26% for a follow-up of 6–11 years [31].

Another explanation for declining mortality might be found in a more restrictive prescription pattern of hormone replacement therapy (HRT). Principal results from the Women`s Health Initiative randomized controlled trial revealed an increased breast cancer rate of about 26% following HRT [32]. Katalinic et al. noticed a simultaneous fall in HRT prescription and breast cancer incidence in Germany, assuming a cause-effect relationship [33].

The overall lower breast cancer mortality in East Germany is connected to the incidence, which is about 35% lower than in West Germany [34]. A reason for this difference is based on differing reproductive histories of East and West German women. It is known that pregnancy plays a protective role in breast cancer development [35, 36]. An early first pregnancy and multiparity are favourable contributors to a reduced breast cancer lifetime risk [37]. A comparison of the fertility patterns for a cohort of 1961 showed that the median age at giving first birth was 27 years in West Germany and 22 years in East Germany [38]. The total number of births in 40-year-old women was 1.62 in West Germany and 1.84 in East Germany for the cohort of 1955.

In the former GDR, women above an age of 65 years were less likely to receive additional treatment beyond mastectomy or primary palliative care. This is indicated by the number of inpatient treatments, which is close to the number of incident cases in this age group. Furthermore, outpatient treatment of elderly patients was less intense (lower cases when compared with the incidence) and often confined to centres that were hard to reach [26]. It can be argued that this disadvantageous situation prevailed until 2007 when the decline in East Germany reached a plateau.

### Prostate cancer

Our findings illustrated a constant decline in prostate cancer mortality. However, research revealed that the age-standardized incidence of prostate cancer cumulated in the year 2007 (Rate: 120.6 per 100,000 inhabitants) and declined in later years (106.7/100,000 inhabitants in 2012), which thus cannot serve as an explanation for our observations [39].

As prostate cancer is rarely affected by life style factors, an increasingly advantageous effect of secondary prevention and/or therapy might account for the observed mortality decline.

According to a German study that used register data from the Munich area, both of these factors contributed to the favourable mortality data. The authors stated that a broader application of the PSA screening resulted in the detection of small tumours and thus a higher proportion of earlier T categories, from capsule-exceeding to capsule-respecting (proportion of T1 increased from 14% to 32%). They also concluded that a change towards more radical therapy regimens (from 20% to 50% from 1990 to 2010) led to improved survival [40].

### Limitations

As a mere ecological study, this research cannot infer any causal relations. The forecasts are based on previous observations, thus future public health interventions or treatment advances will have an additional effect that cannot be modelled in the present study. However, past trends were continuous for all considered cancer types and no leaps were observed that could be attributed to any particular intervention (public health or treatment). Such a continuous time series can well be modelled by means of time series analyses. In this context it must be stated that statistical uncertainty for the majority of forecasts was considerable, as reflected by the wide confidence intervals.

## Conclusion

The overall trends and courses of cancer mortality were similar between East and West Germany, though the existing differences are likely to continue in the near future.

## Additional files

Additional file 1: Details of statistical analyses. (DOCX 27 kb)
Additional file 2: Figure S1. Internal validation. The forecast error was computed as the percentage difference between the forecasted and observed mortality. The bias was computed as the relative average deviation of the forecasted from the observed values. Additionally, prediction intervals of the forecast errors are displayed (confidence intervals of each forecast, in contrast to the mean error which is represented by the bias). (TIF 109 kb)
Additional file 3: Figure S2. Time series of annual mortality rates for death from lung cancer in Germany excluding the early years (1990–1995). Black: East Germany; Grey: West Germany. Solid lines represent the estimates of the forecast with the respective 95% confidence intervals (dashed lines). The time series covers the time between 1995 and 2014. (TIF 109 kb)
Additional file 4: Figure S3. Ratio of annual mortality rates in East and West Germany for death from lung cancer in Germany excluding the early years (1990–1995). The mortality rate ratio was computed as the ratio of the annual age-standardized mortality in East Germany to that in West Germany. The time series covers the time between 1995 and 2014. (TIF 80 kb)
Additional file 5: Figure S4. Time series of annual mortality rates for death from colorectal cancer in Germany excluding the early years (1990–1995). Black: East Germany; Grey: West Germany. Solid lines represent the estimates of the forecast with the respective 95% confidence intervals (dashed lines). The time series covers the time between 1995 and 2014 (TIF 105 kb)
Additional file 6: Figure S5. Ratio of annual mortality rates in East and West Germany for death from colorectal cancer in Germany excluding the early years (1990–1995). The mortality rate ratio was computed as the ratio of the annual age-standardized mortality in East Germany to that in West Germany. The time series covers the time between 1995 and 2014. (TIF 81 kb)
Additional file 7: Figure S6. Time series of annual mortality rates for death from prostate and breast cancer in Germany excluding the early years (1990–1995). Black: East Germany; Grey: West Germany. Solid lines represent the estimates of the forecast with the respective 95% confidence intervals (dashed lines). The time series covers the time between 1995 and 2014 (TIF 106 kb)
Additional file 8: Figure S7. Ratio of annual mortality rates in East and West Germany for death from prostate and breast cancer in Germany excluding the early years (1990–1995). The mortality rate ratio was computed as the ratio of the annual age-standardized mortality in East Germany to that in West Germany. The time series covers the time between 1995 and 2014. (TIF 81 kb)
Additional file 9: Figure S8. Time series of annual mortality rates for death from lung cancer in Berlin. Solid lines represent the estimates of the forecast with the respective 95% confidence intervals (dashed lines). (TIF 94 kb)
Additional file 10: Figure S9. Time series of annual mortality rates for death from colorectal cancer in Berlin. Solid lines represent the estimates of the forecast with the respective 95% confidence intervals (dashed lines). (TIF 91 kb)
Additional file 11: Figure S10. Time series of annual mortality rates for death from prostate and breast cancer in Berlin. Solid lines represent the estimates of the forecast with the respective 95% confidence intervals (dashed lines). (TIF 35 kb)
Additional file 12: Table S1. Annual percentage cancer mortality change in Berlin with 95% confidence intervals. Estimates were computed using a joinpoint analysis with 2000 permutations. (DOCX 16 kb)

## Abbreviations

ACRS: Age and cancer site case-mix standardized relative survival
ARIMA: Autoregressive integrated moving average
CI: Confidence interval
EG: East Germany
FBS: Federal bureau of statistics
FRG: Federal Republic of Germany
GDR: German Democratic Republic
MMR: Mortality rate ratio
NCI: National cancer institute
WG: West Germany.

## References

[1] Bosetti C, Bertuccio P, Malvezzi M, Levi F, Chatenoud L, Negri E, La Vecchia C (2013). Cancer mortality in Europe, 2005–2009, and an overview of trends since 1980. Ann Oncol.

[2] Baili P, Di Salvo F, Marcos-Gragera R, Siesling S, Mallone S, Santaquilani M, Micheli A, Lillini R, Francisci S, Group E-W: Age and case mix-standardised survival for all cancer patients in Europe 1999–2007: Results of EUROCARE-5, a population-based study. Eur J Cancer. 2015;51(15):2120–2129. 10.1016/j.ejca.2015.07.025 26421816

[3] Quaglia A, Lillini R, Mamo C, Ivaldi E, Vercelli M, SEIH (Socio-Economic Indicators HaWG (2013). Socio-economic inequalities: a review of methodological issues and the relationships with cancer survival. Crit Rev Oncol Hematol.

[4] Lillini R, Vercelli M, Quaglia A, Micheli A, Capocaccia R (2011). Use of socio-economic factors and healthcare resources to estimate cancer survival in European countries with partial national cancer registration. Tumori.

[5] Jansen L, Gondos A, Eberle A, Emrich K, Holleczek B, Katalinic A, Brenner H, Group GCSW (2012). Cancer survival in Eastern and Western Germany after the fall of the iron curtain. Eur J Epidemiol.

[6] Helfenstein U (1996). Box-Jenkins modelling in medical research. Stat Methods Med Res.

[7] Box GEP, Jenkins GM (1976). Time series analysis: forecasting and control.

[8] Kim HJ, Fay MP, Feuer EJ, Midthune DN (2000). Permutation tests for joinpoint regression with applications to cancer rates. Stat Med.

[9] Team RC (2014). A Language and Environment for Statistical Computing.

[10] Kibele EUB: Regional mortality differences in Germany. In. Dordrecht: Demographic Research Monographs, Springer; 2012. pp. 14–22.

[11] Luy M: Mortality differences between Western and Eastern Germany before and after Reunification A macro and micro level analysis of developments and responsible factors. Genus. 2004;60(3/4):99–141.

[12] Nolte E, Shkolnikov V, McKee M (2000). Changing mortality patterns in East and West Germany and Poland. II: short-term trends during transition and in the 1990s. J Epidemiol Community Health.

[13] Islami F, Torre LA, Jemal A (2015). Global trends of lung cancer mortality and smoking prevalence. Transl Lung Cancer Res.

[14] John U, Hanke M (2015). Lung cancer mortality and years of potential life lost among males and females over six decades in a country with high smoking prevalence: an observational study. BMC Cancer.

[15] Myrskylä M, Scholz R (2013). Reversing East–west mortality difference among German women, and the role of smoking. Int J Epidemiol.

[16] Malvezzi M, Bertuccio P, Rosso T, Rota M, Levi F, La Vecchia C, Negri E (2015). European cancer mortality predictions for the year 2015: does lung cancer have the highest death rate in EU women?. Ann Oncol.

[17] Bosetti C, Levi F, Rosato V, Bertuccio P, Lucchini F, Negri E, La Vecchia C (2011). Recent trends in colorectal cancer mortality in Europe. Int J Cancer.

[18] Hamza S, Cottet V, Touillon N, Dancourt V, Bonithon-Kopp C, Lepage C, Faivre J (2014). Long-term effect of faecal occult blood screening on incidence and mortality from colorectal cancer. Dig Liver Dis.

[19] Brenner H, Altenhofen L, Kretschmann J, Rösch T, Pox C, Stock C, Hoffmeister M (2015). Trends in Adenoma Detection Rates During the First 10 Years of the German Screening Colonoscopy Program. Gastroenterology.

[20] de Leon MP, Pezzi A, Benatti P, Manenti A, Rossi G, di Gregorio C, Roncucci L (2009). Survival, surgical management and perioperative mortality of colorectal cancer in the 21-year experience of a specialised registry. Int J Colorectal Dis.

[21] Sauer R, Becker H, Hohenberger W, Rödel C, Wittekind C, Fietkau R, Martus P, Tschmelitsch J, Hager E, Hess CF (2004). Preoperative versus postoperative chemoradiotherapy for rectal cancer. N Engl J Med.

[22] Sauer R, Liersch T, Merkel S, Fietkau R, Hohenberger W, Hess C, Becker H, Raab HR, Villanueva MT, Witzigmann H (2012). Preoperative versus postoperative chemoradiotherapy for locally advanced rectal cancer: results of the German CAO/ARO/AIO-94 randomized phase III trial after a median follow-up of 11 years. J Clin Oncol.

[23] Meyerhardt JA, Mayer RJ (2005). Systemic therapy for colorectal cancer. N Engl J Med.

[24] Song M, Garrett WS, Chan AT (2015). Nutrients, foods, and colorectal cancer prevention. Gastroenterology.

[25] Mensink GB, Beitz R (2004). Food and nutrient intake in East and West Germany, 8 years after the reunification--The German Nutrition Survey 1998. Eur J Clin Nutr.

[26] Germany (West). Bundesminister fur Jugend FuG (1993). Indikatoren zum Gesundheitszustand der Bevolkerung in der ehemaligen DDR Herausgeber: Der Bundesminister fur Gesundheit, vol. Bd. 23.

[27] Kaplan HG, Malmgren JA, Atwood MK, Calip GS (2015). Effect of treatment and mammography detection on breast cancer survival over time: 1990–2007. Cancer.

[28] Agarwal S, Pappas L, Neumayer L, Kokeny K, Agarwal J (2014). Effect of breast conservation therapy vs mastectomy on disease-specific survival for early-stage breast cancer. JAMA Surg.

[29] McGale P, Taylor C, Correa C, Cutter D, Duane F, Ewertz M, Gray R, Mannu G, Peto R, Whelan T (2014). Effect of radiotherapy after mastectomy and axillary surgery on 10-year recurrence and 20-year breast cancer mortality: meta-analysis of individual patient data for 8135 women in 22 randomised trials. Lancet.

[30] Darby S, McGale P, Correa C, Taylor C, Arriagada R, Clarke M, Cutter D, Davies C, Ewertz M, Godwin J (2011). Effect of radiotherapy after breast-conserving surgery on 10-year recurrence and 15-year breast cancer death: meta-analysis of individual patient data for 10,801 women in 17 randomised trials. Lancet.

[31] Njor S, Nyström L, Moss S, Paci E, Broeders M, Segnan N, Lynge E, Group EW (2012). Breast cancer mortality in mammographic screening in Europe: a review of incidence-based mortality studies. J Med Screen.

[32] Rossouw JE, Anderson GL, Prentice RL, LaCroix AZ, Kooperberg C, Stefanick ML, Jackson RD, Beresford SA, Howard BV, Johnson KC (2002). Risks and benefits of estrogen plus progestin in healthy postmenopausal women: principal results From the Women's Health Initiative randomized controlled trial. JAMA.

[33] Katalinic A, Lemmer A, Zawinell A, Rawal R, Waldmann A (2009). Trends in hormone therapy and breast cancer incidence - results from the German Network of Cancer Registries. Pathobiology.

[34] Katalinic A, Pritzkuleit R, Waldmann A (2009). Recent Trends in Breast Cancer Incidence and Mortality in Germany. Breast Care (Basel).

[35] Russo J, Moral R, Balogh GA, Mailo D, Russo IH (2005). The protective role of pregnancy in breast cancer. Breast Cancer Res.

[36] Britt K, Ashworth A, Smalley M (2007). Pregnancy and the risk of breast cancer. Endocr Relat Cancer.

[37] Ursin G, Bernstein L, Lord SJ, Karim R, Deapen D, Press MF, Daling JR, Norman SA, Liff JM, Marchbanks PA (2005). Reproductive factors and subtypes of breast cancer defined by hormone receptor and histology. Br J Cancer.

[38] Kreyenfeld M: Crisis or Adaptation – Reconsidered: A Comparison of East and West German Fertility Patterns in the First Six Years after the ‘Wende'. European Journal of Population / Revue européenne de Démographie. 2003;19(3):303–329.

[39] Zentrum für Krebsregisterdaten [http://www.krebsdaten.de/Krebs/SiteGlobals/Forms/Datenbankabfrage/datenbankabfrage_stufe1_form.html]. Accessed 11 May 2016.

[40] Dörr M, Hölzel D, Schubert-Fritschle G, Engel J, Schlesinger-Raab A (2015). Changes in prognostic and therapeutic parameters in prostate cancer from an epidemiological view over 20 years. Oncol Res Treat.

