# Supplementary material for: Cancer mortality in former East and West Germany: a story of unification?
Source: BMC Cancer. 2017 Feb 2;17:94. doi: 10.1186/s12885-017-3086-y (PMC5288858; doi:10.1186/s12885-017-3086-y)
Supplement: Additional file 1: — Details of statistical analyses. (DOCX 27 kb) [file 12885_2017_3086_MOESM1_ESM.docx]

**Appendix**

As the methods used in our analysis are rarely applied in medical research, we provide a short overview of the essentials in this Appendix, a comprehensive account can be found in Helfenstein [1].

A time series *n_t_* can in most cases (and also in our work) be adequately modelled by a SARIMA-model (seasonal ARIMA model), which is expressed by ARIMA(p,d,q). Basically, an ARIMA model consists of three terms which model the order of the AR (autoregressive, *p*), the I (integrated, *d*), and the MA (moving average, *q*) part of *n_t_*.

**(1) The AR part:**

For convenience, we start with an AR(1) model, that is, an AR model with *p*=1. This can be interpreted like an ordinary linear regression equation with *y_t_* as the response, *φ* as the regression parameter for the single covariate *y_t-1_*_,_ and a normally distributed random error $\epsilon_{t}$.

| $y_{t}=\phi y_{t-1}+\epsilon_{t}$ | $\epsilon_{t}\sim N(0, \sigma^{2})$ |
| --- | --- |
|  |  |

The current value of the model, *y_t_* is the sum of the previous value *y_t-1_* (multiplied by *φ*) and the random error. The association between *y_t_* and *y_t-1_* is controlled by the AR(1) parameter *φ:* the larger *φ*, the higher is the correlation between *y_t_* and *y_t-1_*. The idea of regressing the current value on its own predecessor explains the term autoregressive for this model. Autoregressive models of higher orders (AR(*p*) models) are straightforward extensions of the AR(1) process, including the *p* previous values of the process in the model equation.

| $y_{t}={\phi_{1}y}_{t-1}+{\phi_{2}y}_{t-2} +\ldots+{\phi_{p}y}_{t-p}+\epsilon_{t}$ | $\epsilon_{t}\sim N(0, \sigma^{2})$ |
| --- | --- |

|  |
| --- |

**(2) The MA part:**

The idea of an MA model is similar to that of an AR model; however, now the current value of the time series, *y_t_*, is assumed to depend only on random fluctuations. If random fluctuations on the same day $\epsilon_{t}$, and on the day before $\epsilon_{t-1}$ are taken into account, a MA(1) model is defined.

| $y_{t}=\epsilon_{t}+\theta\epsilon_{t-1}$ | $\epsilon_{i}\sim N(0, \sigma^{2})$ |
| --- | --- |

Higher orders q of an MA model are straightforwardly defined as

| $y_{t}=\epsilon_{t}+\theta\epsilon_{t-1}+...+\theta\epsilon_{t-q}$ | $\epsilon_{i}\sim N(0, \sigma^{2})$ |
| --- | --- |

**(3) The I part**

A time series is said to be stationary, if the mean of the time series does not depend upon time, but is constant throughout the complete time course. For a valid intervention analysis, the noise series n_t_ has to be stationary. The easiest way to achieve stationarity is by differentiating the time series by the preceding value s time points ago, where s is the length of the period. Further differentiations with different lags are possible (with d measuring the number of differentiations), but were not necessary in our case.

Actual model fitting thus involves finding the optimal orders of the ARIMA model, the respective parameters, and the parameter of actual interest, the pulse w_0_. Box/Jenkins [2] proposed an algorithm for this model identification which is frequently used in applied research. This algorithm consists of four steps:

**(1) Make the original time series (y_t_) stationary**

Stationarity can be checked via the Dickey-Fuller test. An underlying trend or seasonality is assessable via the empirical autocorrelation and partial autocorrelation functions of *y_t_* at various lags. The autocorrelation at lag *k* is the correlation of the value y_t_ and its predecessors y_t-k_. The partial autocorrelation at lag *k* adjusts for the influence of time points lying between the value *y_t_* and its predecessor *y_t-k_*, leaving only the adjusted correlation between the two values.

**(2) Find a preliminary order of the model**

In the second step, a preliminary order of the ARIMA(*p*,0,*q*) model (that is, p,q) is identified by again referring to the autocorrelation functions.

**(3) Estimate the coefficients of the model**

The coefficients *θ*, *φ*, *Φ* and *Ω* are estimated by maximum likelihood.

**(4) Check the model by assessing the autocorrelations of the residuals**

As a final step, the adequacy of the model from step (3) has to be evaluated, which is done by demanding no relevant autocorrelations of the residuals. If there are no autocorrelations, the model can be regarded as properly modeling the noise series *n_t_*. Finally, all coefficients (*θ*, *φ*, *Φ* and *Ω*) from the noise series are estimated again by maximum likelihood, but now simultaneously with the pulse w_0_. Rinne/Specht [3] suggest a maximum lag of $K\approx2\sqrt{T}$ (in our case the total time points are 35 and 25 years, respectively) when the residuals are checked for autocorrelations as implemented in the method described by Box/Jenkins. Therefore, a lag of 6 and 5 years, respectively, was considered for model checking.

Considering the primary purpose of this paper, the forecast was finally checked for plausibility.

Finally, we identified the following ARIMA models which are given in Table A1.

*Table A2: Selected ARIMA models based on the Box/Jenkins approach*

| Entity | Order of the ARIMA model |
| --- | --- |
| Lung Cancer (Men, East-Germany) | ARIMA(1,1,1) |
| Lung Cancer (Men, West-Germany) | ARIMA(3,1,0) |
| Lung Cancer (Women, East-Germany) | ARIMA(1,1,1) |
| Lung Cancer (Women, West-Germany) | ARIMA(3,1,0) |
|  |  |
| Colorectal Cancer (Men, East-Germany) | ARIMA(1,1,1) |
| Colorectal Cancer (Men, West-Germany) | ARIMA(1,1,2) |
| Colorectal Cancer (Women, East-Germany) | ARIMA(1,1,1) |
| Colorectal Cancer (Women, West-Germany) | ARIMA(1,1,1) |
|  |  |
| Prostate Cancer (Men, East-Germany) | ARIMA(1,1,1) |
| Prostate Cancer (Men, West-Germany) | ARIMA(1,1,1) |
| Breast Cancer (Women, East-Germany) | ARIMA(1,1,1) |
| Breast Cancer (Women, West-Germany) | ARIMA(1,1,1) |

1. Helfenstein U: Box-Jenkins modelling in medical research. Stat Methods Med Res 1996, 5(1):3-22.
2. Box GEP, Jenkins GM: Time series analysis: forecasting and control. In. San Francisco: Holden-Day; 1976.
3. Rinne H. 2002. *Zeitreihen*. *Statistische Modellierung, Schätzung und Prognose.* München: Vahlen
