# Supplementary figures and images for: Cancer mortality in former East and West Germany: a story of unification?
Source: BMC Cancer. 2017 Feb 2;17:94. doi: 10.1186/s12885-017-3086-y (PMC5288858; doi:10.1186/s12885-017-3086-y)

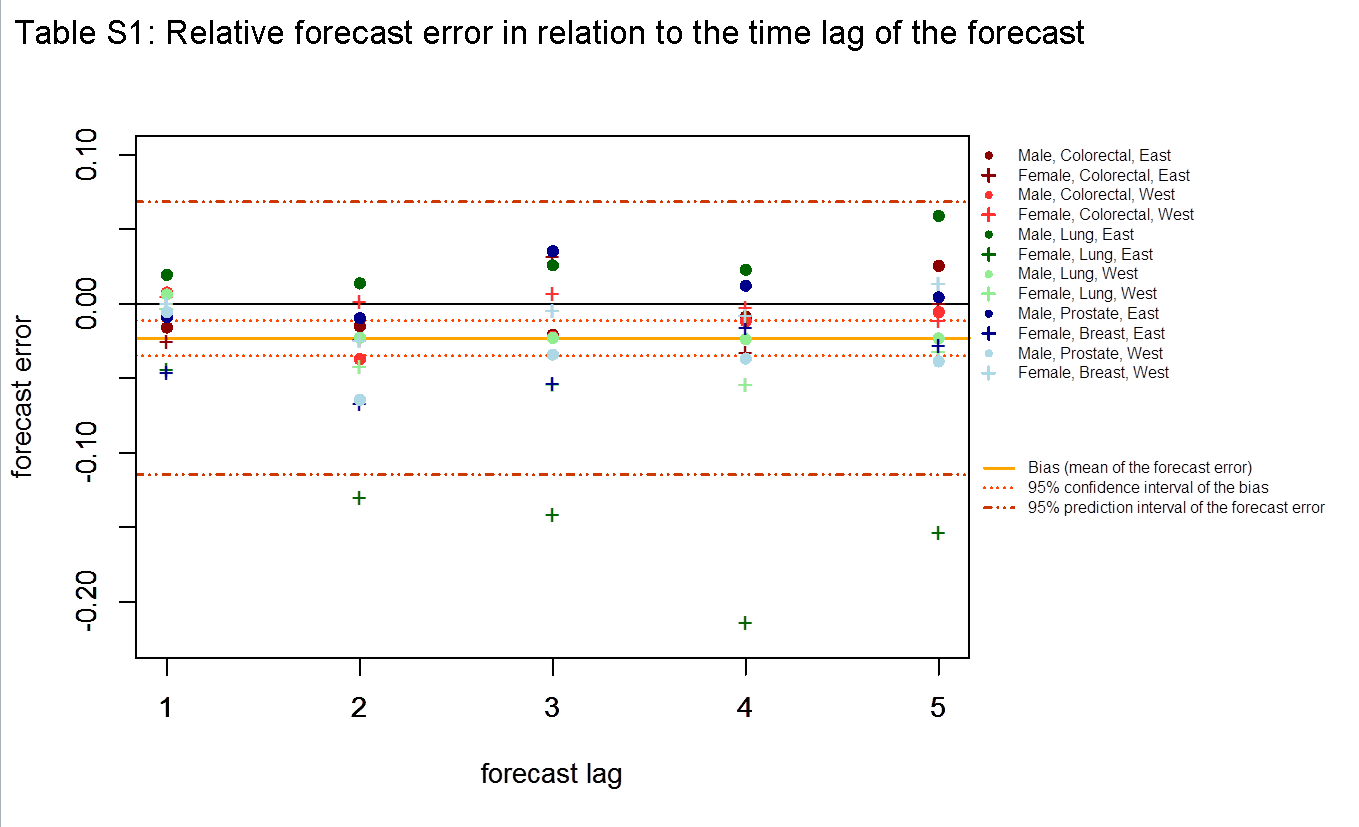

Supplement: Additional file 2: Figure S1. — Internal validation. The forecast error was computed as the percentage difference between the forecasted and observed mortality. The bias was computed as the relative average deviation of the forecasted from the observed values. Additionally, prediction intervals of the forecast errors are displayed (confidence intervals of each forecast, in contrast to the mean error which is represented by the bias). (TIF 109 kb) [file 12885_2017_3086_MOESM2_ESM.tif]

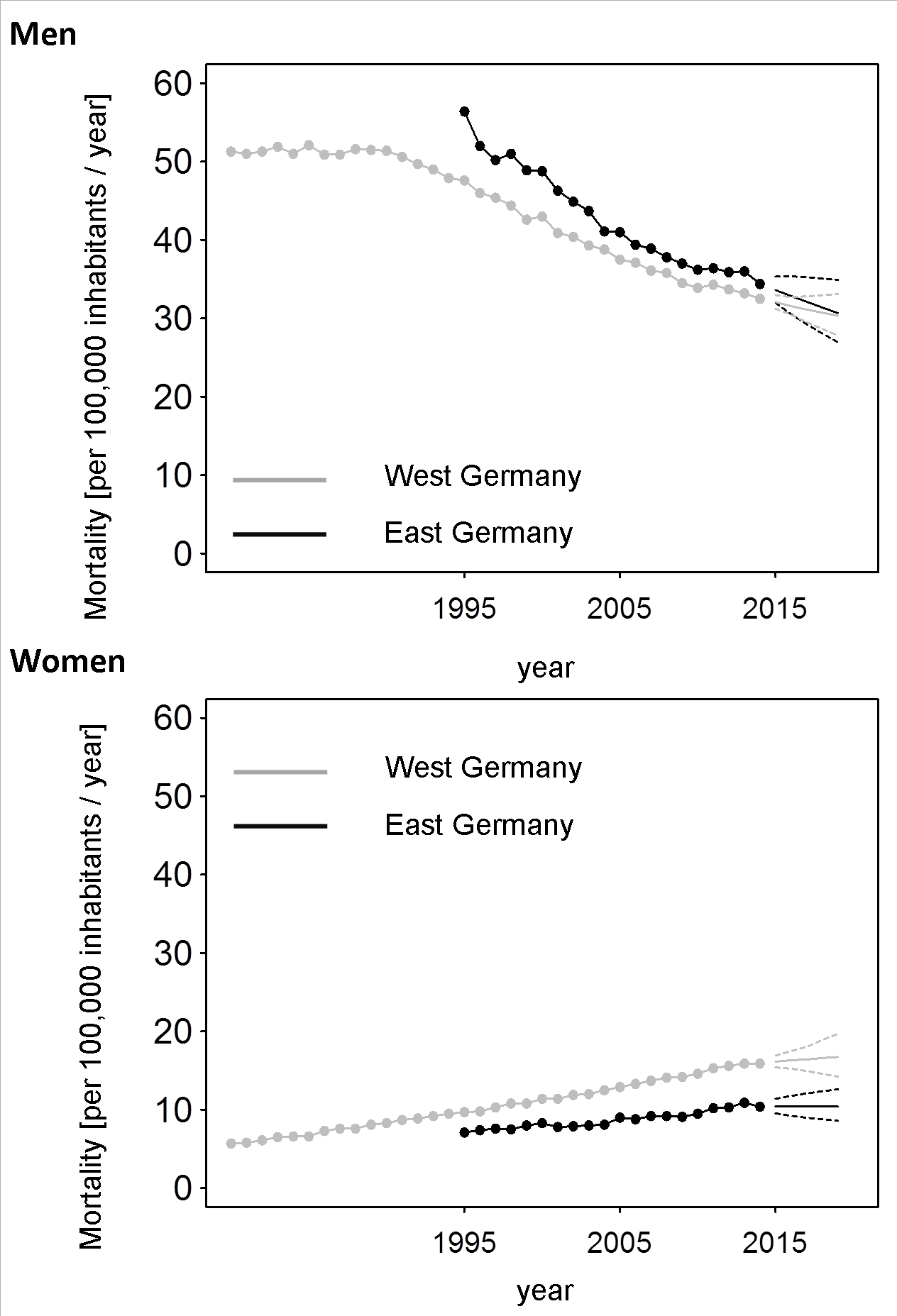

Supplement: Additional file 3: Figure S2. — Time series of annual mortality rates for death from lung cancer in Germany excluding the early years (1990–1995). Black: East Germany; Grey: West Germany. Solid lines represent the estimates of the forecast with the respective 95% confidence intervals (dashed lines). The time series covers the time between 1995 and 2014. (TIF 109 kb) [file 12885_2017_3086_MOESM3_ESM.tif]

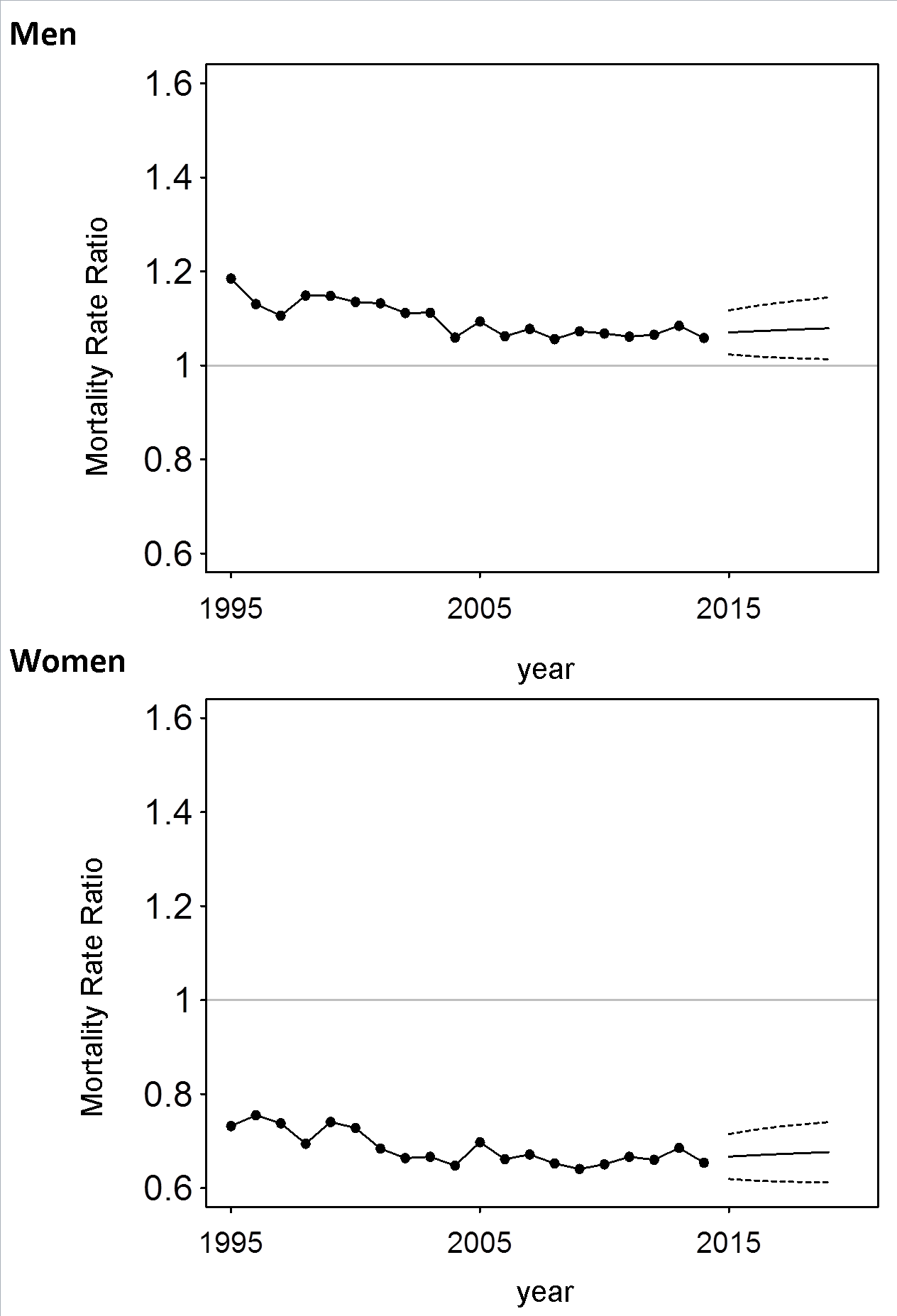

Supplement: Additional file 4: Figure S3. — Ratio of annual mortality rates in East and West Germany for death from lung cancer in Germany excluding the early years (1990–1995). The mortality rate ratio was computed as the ratio of the annual age-standardized mortality in East Germany to that in West Germany. The time series covers the time between 1995 and 2014. (TIF 80 kb) [file 12885_2017_3086_MOESM4_ESM.tif]

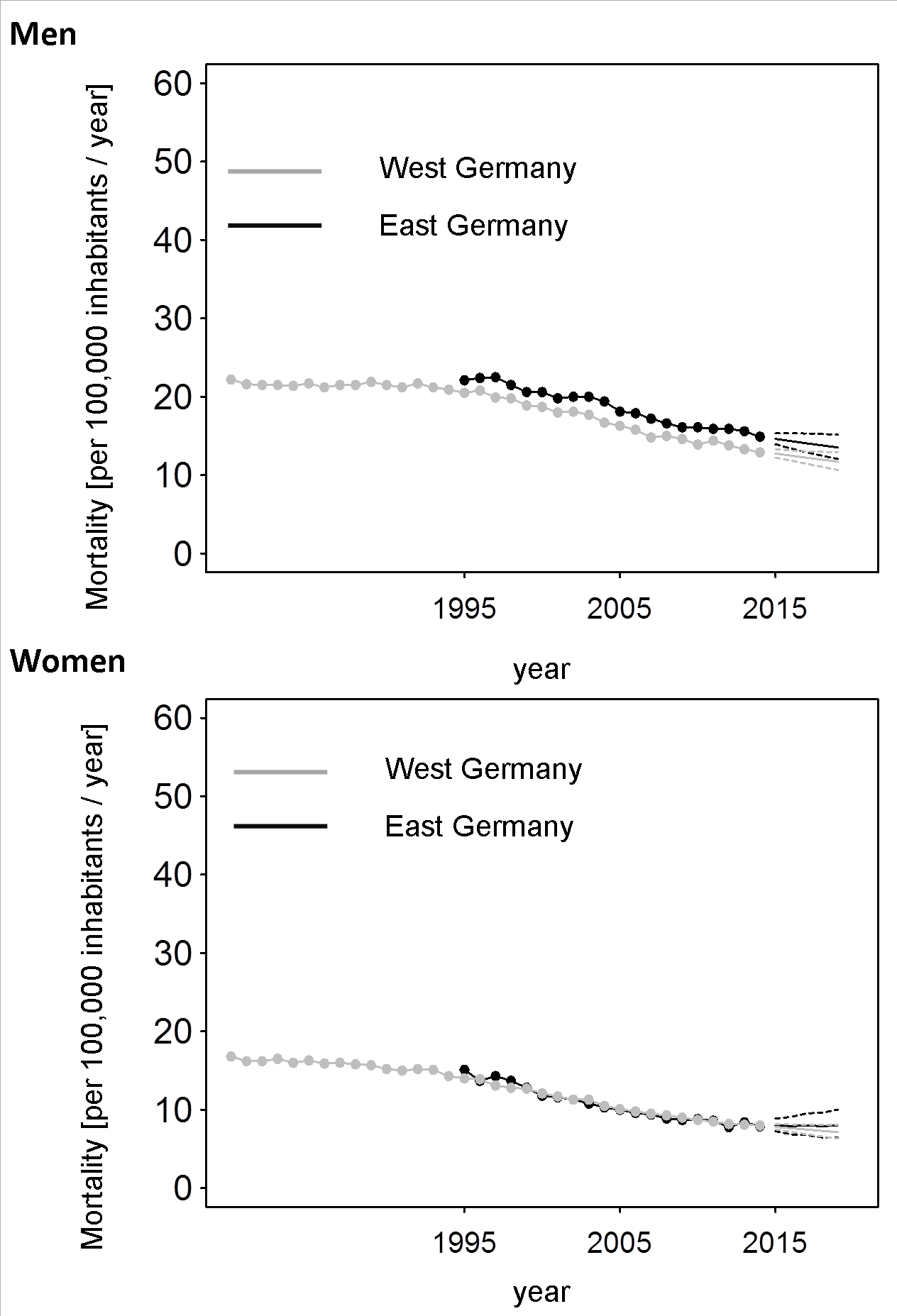

Supplement: Additional file 5: Figure S4. — Time series of annual mortality rates for death from colorectal cancer in Germany excluding the early years (1990–1995). Black: East Germany; Grey: West Germany. Solid lines represent the estimates of the forecast with the respective 95% confidence intervals (dashed lines). The time series covers the time between 1995 and 2014 (TIF 105 kb) [file 12885_2017_3086_MOESM5_ESM.tif]

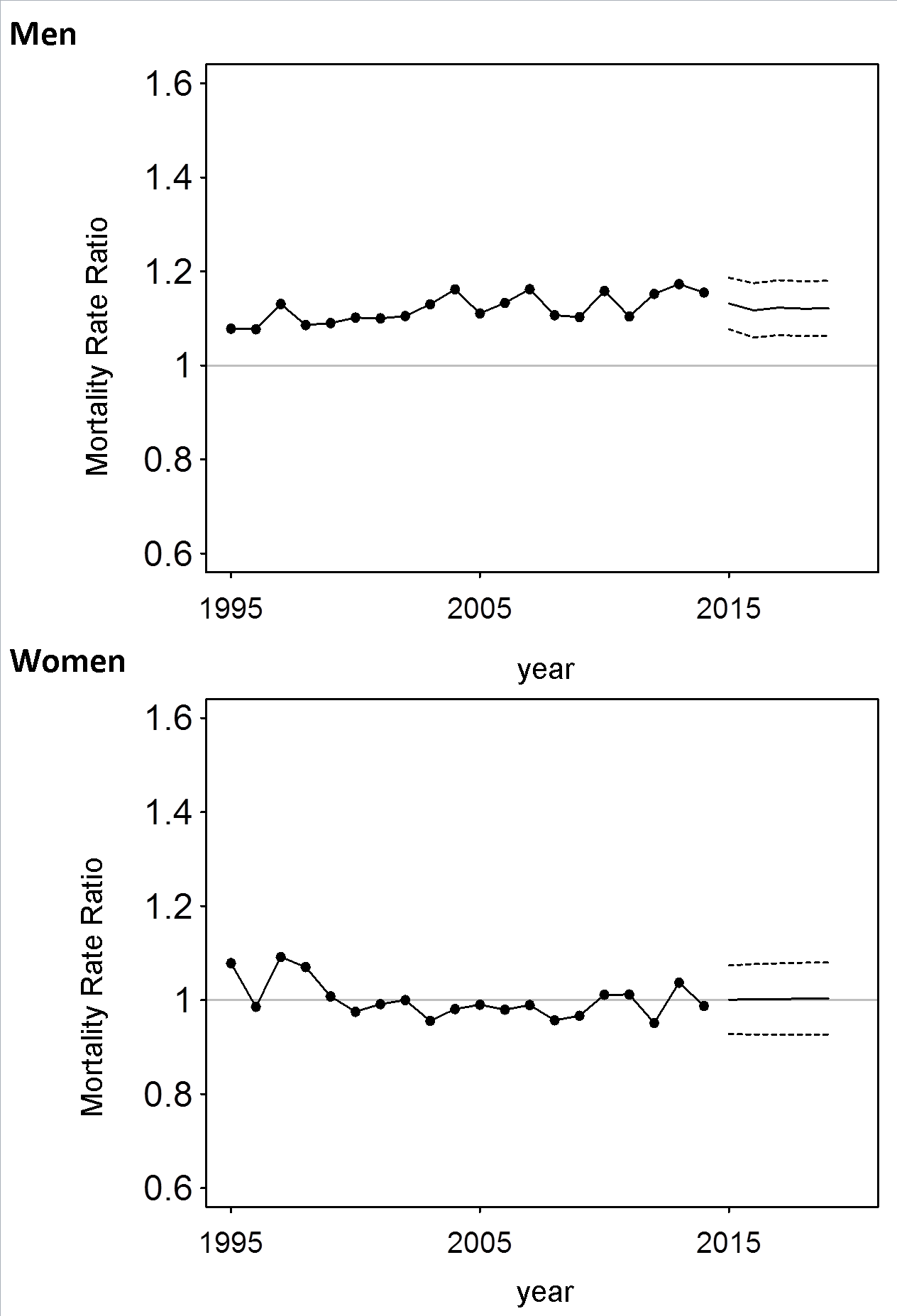

Supplement: Additional file 6: Figure S5. — Ratio of annual mortality rates in East and West Germany for death from colorectal cancer in Germany excluding the early years (1990–1995). The mortality rate ratio was computed as the ratio of the annual age-standardized mortality in East Germany to that in West Germany. The time series covers the time between 1995 and 2014. (TIF 81 kb) [file 12885_2017_3086_MOESM6_ESM.tif]

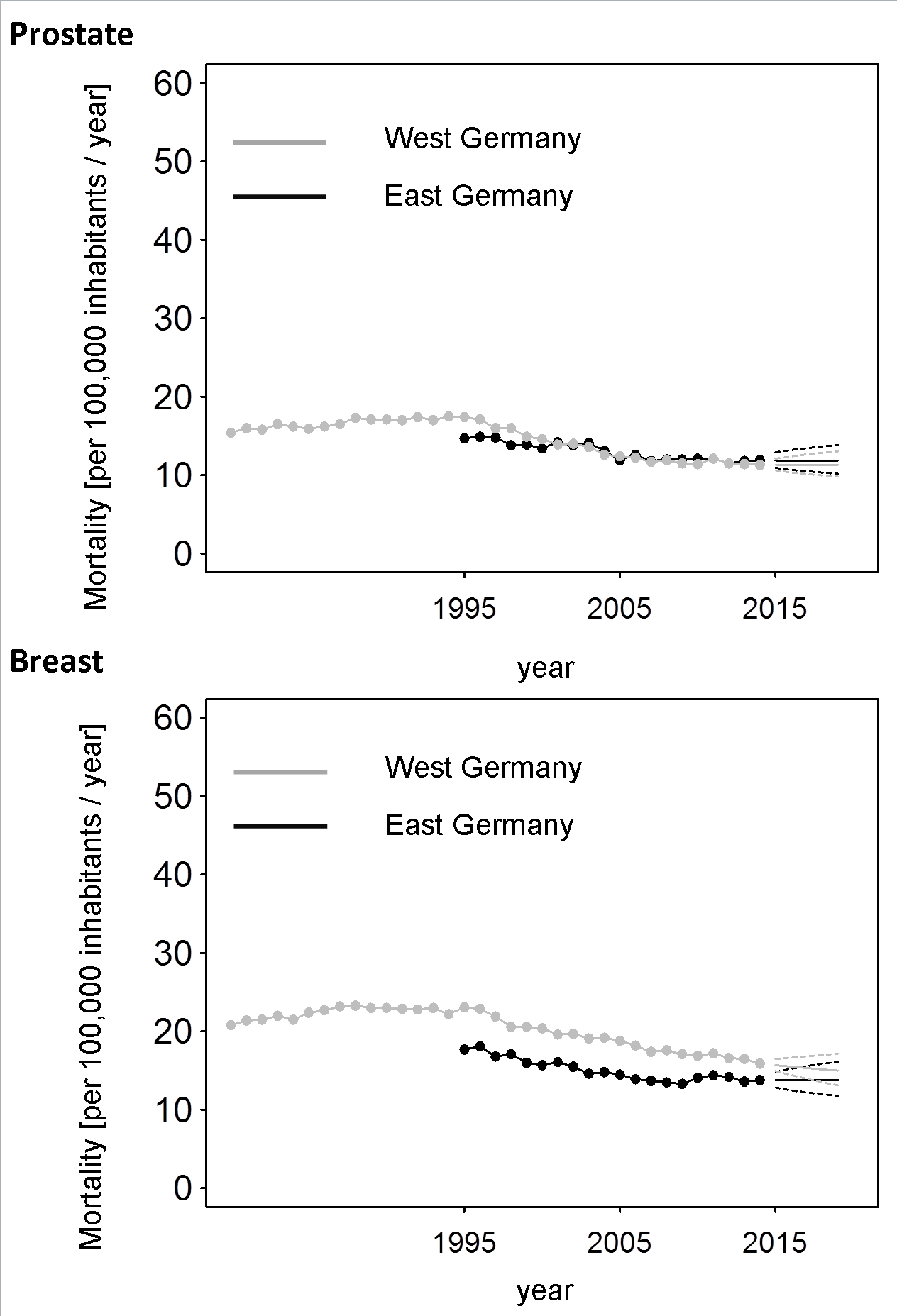

Supplement: Additional file 7: Figure S6. — Time series of annual mortality rates for death from prostate and breast cancer in Germany excluding the early years (1990–1995). Black: East Germany; Grey: West Germany. Solid lines represent the estimates of the forecast with the respective 95% confidence intervals (dashed lines). The time series covers the time between 1995 and 2014 (TIF 106 kb) [file 12885_2017_3086_MOESM7_ESM.tif]

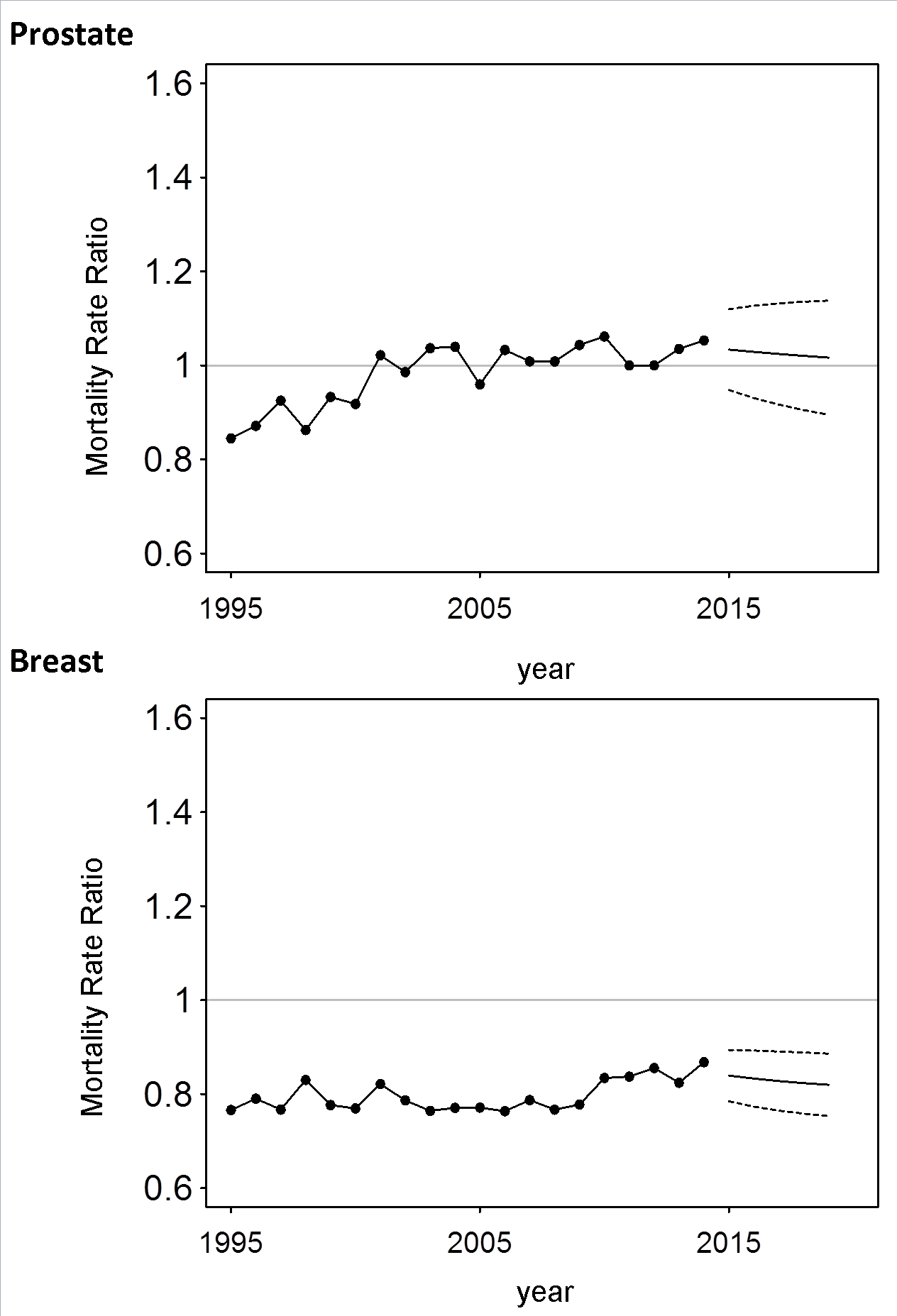

Supplement: Additional file 8: Figure S7. — Ratio of annual mortality rates in East and West Germany for death from prostate and breast cancer in Germany excluding the early years (1990–1995). The mortality rate ratio was computed as the ratio of the annual age-standardized mortality in East Germany to that in West Germany. The time series covers the time between 1995 and 2014. (TIF 81 kb) [file 12885_2017_3086_MOESM8_ESM.tif]

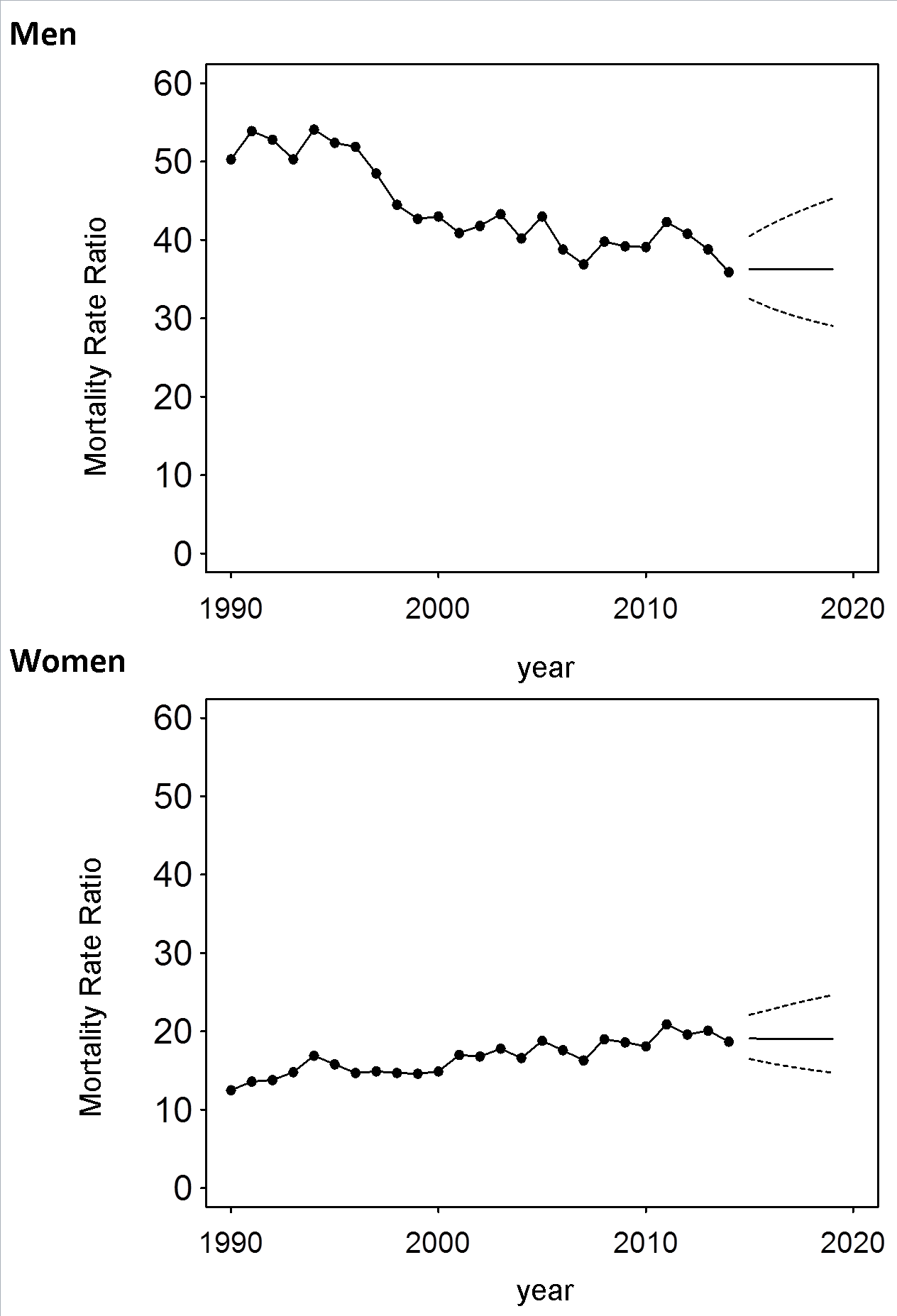

Supplement: Additional file 9: Figure S8. — Time series of annual mortality rates for death from lung cancer in Berlin. Solid lines represent the estimates of the forecast with the respective 95% confidence intervals (dashed lines). (TIF 94 kb) [file 12885_2017_3086_MOESM9_ESM.tif]

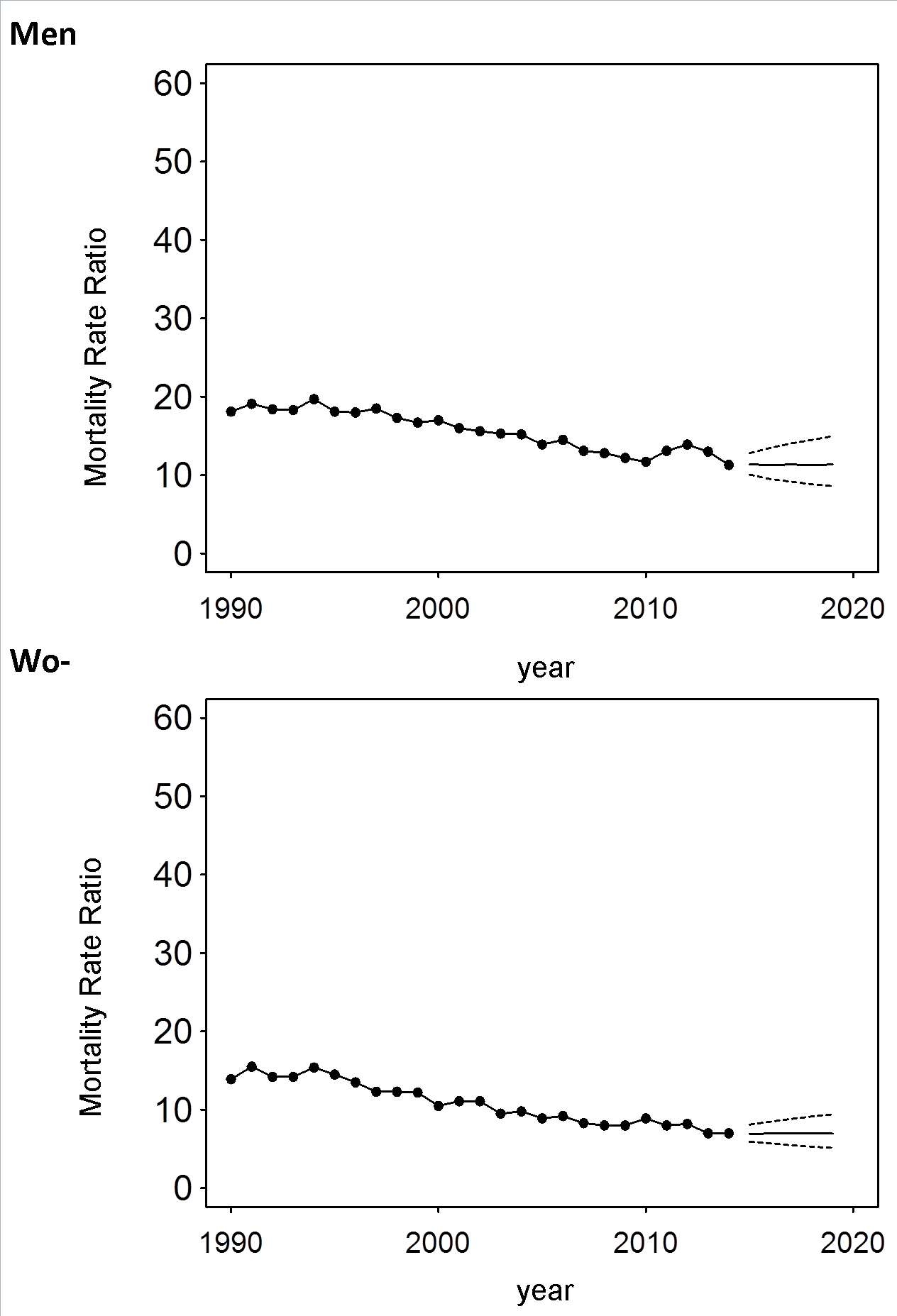

Supplement: Additional file 10: Figure S9. — Time series of annual mortality rates for death from colorectal cancer in Berlin. Solid lines represent the estimates of the forecast with the respective 95% confidence intervals (dashed lines). (TIF 91 kb) [file 12885_2017_3086_MOESM10_ESM.tif]

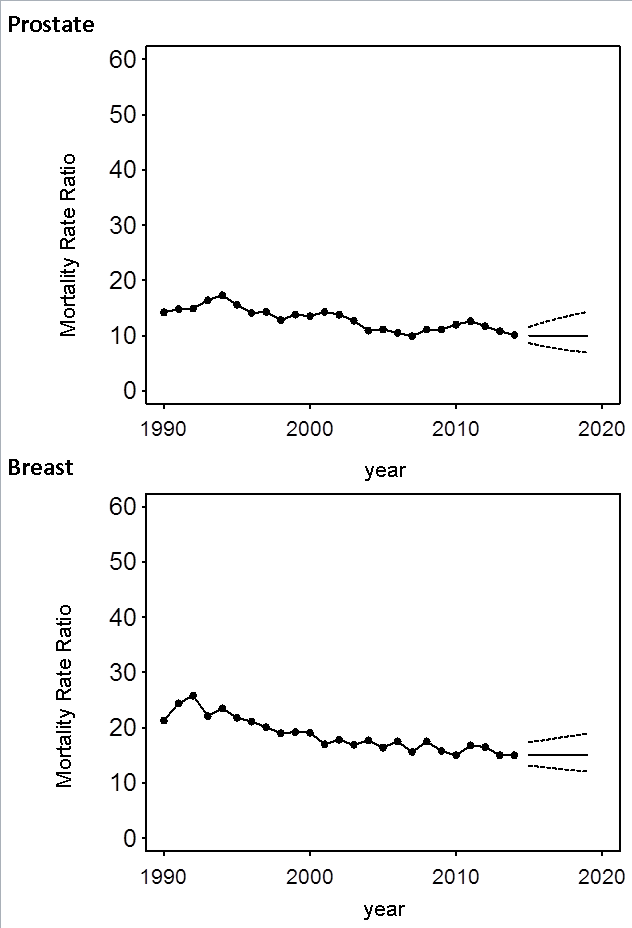

Supplement: Additional file 11: Figure S10. — Time series of annual mortality rates for death from prostate and breast cancer in Berlin. Solid lines represent the estimates of the forecast with the respective 95% confidence intervals (dashed lines). (TIF 35 kb) [file 12885_2017_3086_MOESM11_ESM.tif]
