# Supplementary material for: Cancer mortality in former East and West Germany: a story of unification?
Source: BMC Cancer. 2017 Feb 2;17:94. doi: 10.1186/s12885-017-3086-y (PMC5288858; doi:10.1186/s12885-017-3086-y)
Supplement: Additional file 12: Table S1. — Annual percentage cancer mortality change in Berlin with 95% confidence intervals. Estimates were computed using a joinpoint analysis with 2000 permutations. (DOCX 16 kb) [file 12885_2017_3086_MOESM12_ESM.docx]

| Men |  |  |  | |  | |  | |
| --- | --- | --- | --- | --- | --- | --- | --- | --- |
|  |  | Slope 1 | Slope 2 | Slope 3 | | Slope 4 | |  |
| Lung | **Year** | **1980-1996** | **1991-1999** | **1999-2014** | | **-** | |  |
|  | Estimate | 0.1 [-2.1, 2.4] | -6.7 [-18.3, 6.5] | -0.7 [-1.2, -0.1] | | - | |  |
| Prostate | **Year** | **1990-1994** | **1994-2007** | **2007-2011** | | **2011-2014** | |  |
|  | Estimate | 3.6 [-2,1, 9.6] | -3.4 [-4.4, -2.4] | 4.8 [-4.2, 14.5] | | -7.0 [-14.9, 1.7] | |  |
| Colon/Rectum | **Year** | **1980-1992** | **1992-1995** | **1992-1995** | | **1992-1995** | |  |
|  | Estimate | 9.0 [-0.6, 19.7] | -3.4 [-12.0, 6.0] | 3.4 [2.8, 4.1] | | 0.2 [-1.1, 1.4] | |  |
| Women |  |  |  |  | |  | |  |
| Lung | **Year** | **1990-2014** | - | - | | - | |  |
|  | Estimate | 1.6 [1.3, 2.0] | - | - | | - | |  |
| Breast | **Year** | **1990-1992** | **1992-2001** | **2001-2014** | | - | |  |
|  | Estimate | 6.3 [-6.6, 21.1] | -3.7 [-5.0, -2.3] | -1.0 [-1.7, -0.4] | | - | |  |
| Colon/Rectum | **Year** | **1990-2014** | - | - | | - | |  |
|  | Estimate | 3.4 [3.0, 3.8] | - | - | | - | |  |
